# Editorial: Exploring unconventional T cells and CAR-T-cells in cancer immunotherapy

**DOI:** 10.3389/fimmu.2026.1900363

**Published:** 2026-06-11

**Authors:** Laura Patrussi, Chenran Zhang

**Affiliations:** 1 Department of Life Sciences, University of Siena, Siena, Italy; 2 Laboratory of Human Carcinogenesis, Center of Cancer Research, National Cancer Institute, Bethesda, MD, United States

**Keywords:** CAR T cell, gamma delta T (γδ T) cells, iNKT (invariant natural killer T cell), MAIT (mucosal-associated invariant T) cell, tumor microenvironment - TME, unconventional T cell

Cancer immunotherapy is entering a new phase in which the stimulation of conventional adaptive immunity is increasingly complemented by the exploitation of unconventional T cells (UCTs) and by the refinement of chimeric antigen receptor (CAR)-based cellular therapies. Unlike conventional αβ T cells, UCTs, including γδ T cells, invariant Natural Killer T (iNKT) cells, and mucosal-associated invariant T (MAIT) cells, recognize non-peptide antigens, stress-associated molecules, and metabolic intermediates in an MHC-independent or non-classical MHC-restricted manner. These properties position them at the interface between innate and adaptive immunity and make them particularly attractive candidates for next-generation cancer immunotherapy.

**Figure 1:**
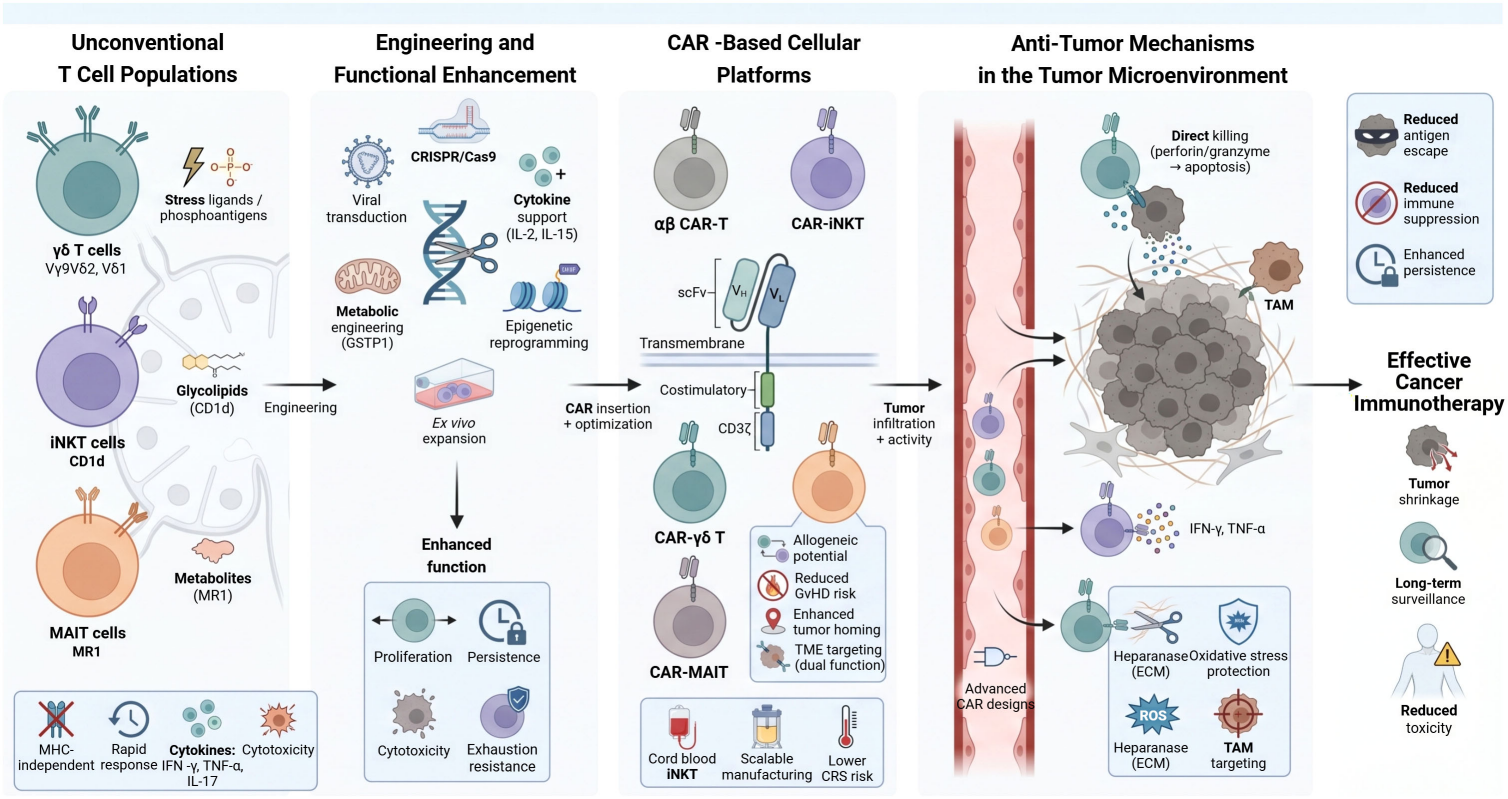
Next-generation cancer immunotherapy strategies using UCTs and CAR-based engineering. The illustration highlights the workflow for developing advanced cellular therapies, starting from the identification of UCT populations (γδ T cells, iNKT cells, and MAIT cells) and their specific recognition of non-peptide antigens. These cells undergo engineering and functional enhancement through techniques such as CRISPR/Cas9, metabolic reprogramming (e.g., GSTP1), and cytokine support to improve persistence and cytotoxicity. The figure showcases various CAR-based platforms, emphasizing the “off-the-shelf” potential of allogeneic iNKT cells and the development of logic-gated CAR designs. Finally, it depicts the anti-tumor mechanisms within the TME, including direct killing, targeting of TAMs, and the use of matrix-remodeling enzymes like heparanase to enhance infiltration and overcome immunosuppression. CAR, chimeric antigen receptor; CRS, cytokine release syndrome; ECM, extracellular matrix; GvHD, graft-versus-host disease; iNKT, invariant natural killer T cells; MAIT, mucosal-associated invariant T cells; ROS, reactive oxygen species; TAM, tumor-associated macrophage; TME, tumor microenvironment; UCT, unconventional T cell. Created with BioRender.com by Laura Patrussi.

This Research Topic highlights the remarkable heterogeneity, functional plasticity, and translational potential of UCTs across solid and hematological malignancies, while also addressing the engineering strategies required to overcome the immunosuppressive tumor microenvironment (TME). Collectively, the contributions illustrate how advances in single-cell technologies, multi-omic profiling, and synthetic immunology are reshaping our understanding of these lymphocyte populations and accelerating their clinical application. The integration of these diverse strategies and cell populations into next-generation immunotherapies is schematically represented in **Figure 1**.

A central theme emerging from this Research Topic is the profound phenotypic and functional diversity of γδ T cells. In human colorectal cancer (CRC), Vδ1^+^ and Vδ2^+^ subsets are generally associated with cytotoxic and anti-tumoral activities mediated by IFN-γ and TNF-α production. However, under specific inflammatory conditions, γδ T cells may also acquire IL-17-producing phenotypes that promote tumor progression, angiogenesis, and immune suppression. These observations underscore the importance of the local cytokine milieu in shaping γδ T-cell behavior and reveal the dual nature of these cells within the TME.

MAIT cells similarly display remarkable functional plasticity. Several studies included in this Topic demonstrate that aging and chronic inflammatory signals profoundly remodel MAIT-cell transcriptional programs. In particular, aging promotes a shift from a MAIT17 phenotype, characterized by IL-17 production and tissue-protective functions, toward a MAIT1 phenotype enriched in IFN-γ-related programs. This transition appears tightly regulated by cytokines such as IL-7, IL-12, IL-18, and TGF-β, together with the transcription factor RORγt. Importantly, alterations in MAIT-cell polarization correlate with disease outcome in colorectal adenocarcinoma, suggesting that cytokine-driven MAIT-cell plasticity may directly influence tumor progression and patient prognosis. These findings further support the concept that UCTs are not fixed effector populations, but highly adaptable immune cells continuously shaped by the inflammatory and metabolic landscape of tumors.

The epigenetic flexibility of iNKT cells represents another important aspect discussed in this Research Topic. Repetitive antigenic stimulation can induce extensive chromatin remodeling and gene de-methylation, leading to the acquisition of regulatory-like phenotypes. Such reprogramming highlights both the therapeutic versatility and the potential exhaustion-related vulnerabilities of iNKT cells during chronic immune activation. Understanding the molecular mechanisms controlling these transitions will be critical for designing durable and effective cell-based therapies.

Beyond human studies, this Research Topic also emphasizes the value of comparative immunology. Investigations in porcine, feline, and canine systems reveal conserved UCT transcriptional programs and cytotoxic mechanisms across species. In dogs, for example, distinct double-negative T-cell subsets exhibiting potent immunoregulatory activity were identified, providing translational insights into immune tolerance and inflammation control. These comparative approaches broaden our understanding of UCT biology and may contribute to the development of more predictive preclinical models for cancer immunotherapy.

The integration of UCTs into CAR platforms represents one of the most promising translational directions highlighted in this Research Topic. Conventional autologous CAR-T cell therapies have achieved remarkable success in hematological malignancies but remain limited by manufacturing complexity, high costs, inter-patient variability, and the risk of graft-versus-host disease (GvHD) in allogeneic settings. In this context, iNKT cells have emerged as highly attractive candidates for “off-the-shelf” allogeneic platforms. Cord blood-derived iNKT cells combine intrinsic anti-tumor activity with a naturally low alloreactive potential, substantially reducing the risk of GvHD.

Importantly, engineered iNKT products display several additional advantages over conventional αβ CAR-T cells, including inter-donor homogeneity, robust ex vivo expansion, and a cytokine profile enriched in IL-4 and IL-10. This Th2-biased signature may help limit severe cytokine release syndrome (CRS) while preserving anti-tumor efficacy. Moreover, iNKT cells possess the unique ability to simultaneously target tumor cells and immunosuppressive components of the TME, including tumor-associated macrophages. These properties make allogeneic iNKT-based CAR therapies particularly appealing for scalable and broadly accessible cellular immunotherapy approaches.

A major barrier to CAR-T cell therapy in solid tumors is the hostile and metabolically restrictive TME. Oxidative stress, nutrient deprivation, chronic antigen exposure, and inhibitory cytokines collectively drive T cell dysfunction and exhaustion. In this context, metabolic engineering strategies are emerging as powerful tools to enhance CAR-T cell fitness. One study identifies glutathione S-transferase-pi 1 (GSTP1) as a key regulator of redox homeostasis in CAR-T cells. By reducing reactive oxygen species accumulation, GSTP1 overexpression improves CAR-T cell proliferation, persistence, and cytotoxic activity in lymphoma models, highlighting the importance of metabolic adaptation in sustaining anti-tumor responses.

The complexity of the TME is particularly evident in solid tumors such as breast cancer and pancreatic ductal adenocarcinoma, where dense stromal barriers, antigen heterogeneity, and poor immune infiltration severely limit therapeutic efficacy. To overcome these obstacles, innovative approaches including dual-target CARs, logic-gated CAR constructs, and matrix-remodeling strategies are being developed. Engineering CAR-T cells to express heparanase, for instance, may facilitate penetration through extracellular matrix components and improve tumor infiltration.

Another particularly exciting area explored in this Research Topic is the use of antibodies targeting BTN3A to selectively activate Vγ9Vδ2 T cells. BTN3A agonistic antibodies such as ICT01 induce conformational changes that trigger phosphoantigen-dependent γδ T-cell activation, promoting rapid cytotoxic responses against tumor cells. Compared with conventional CD3-engaging antibodies, this strategy may offer a more favorable safety profile by selectively activating pre-existing anti-tumoral γδ T-cell populations while limiting widespread polyclonal T-cell activation. Early clinical data further suggest that BTN3A-targeting antibodies may synergize with immune checkpoint inhibitors and could help overcome resistance in immunologically “cold” tumors. Combination approaches with low-dose IL-2 or IL-15 are also under investigation to enhance γδ T-cell expansion and persistence while carefully monitoring the risk of activation-induced exhaustion.

As the potency of engineered cellular therapies increases, the management of treatment-related toxicities becomes increasingly important. CRS and immune effector cell-associated neurotoxicity syndrome remain major complications of CAR-T cell therapy. This Research Topic discusses therapeutic plasma exchange as a potential rescue strategy for severe corticosteroid-refractory CRS, capable of rapidly reducing circulating inflammatory cytokines such as IL-6 and TNF-α. In parallel, the development of logic-gated CAR designs incorporating AND, OR, and NOT circuits offers promising opportunities to improve tumor specificity and minimize on-target/off-tumor toxicity.

Collectively, the studies gathered in this Research Topic position UCTs as both biologically diverse effector cells and adaptable platforms for next-generation cancer immunotherapy. The integration of high-dimensional single-cell technologies, epigenetic profiling, and synthetic engineering approaches is rapidly expanding our capacity to manipulate these cells for therapeutic benefit.

These advances highlight three major opportunities for the field: first, to define when UCTs exert anti-tumoral versus tumor-promoting functions; second, to engineer UCT-based therapies with improved persistence, tissue infiltration, and safety; and third, to combine cellular and antibody-based approaches with rational strategies to overcome TME-mediated suppression. By combining the unique antigen-recognition properties of UCTs with the precision and scalability of CAR engineering, the field is moving closer to overcoming major barriers such as antigen escape, TME-mediated suppression, and treatment-related toxicities. Continued interdisciplinary efforts integrating immunology, cancer biology, bioengineering, and clinical translation will be essential for converting these promising approaches into durable, safe, and broadly accessible therapies for patients with cancer.

